# Viroids, and the Legacy of Ricardo Flores (1947–2020)

**DOI:** 10.3390/cells10102570

**Published:** 2021-09-28

**Authors:** Ahmed Hadidi, John W. Randles

**Affiliations:** 1 U.S. Department of Agriculture, Agricultural Research Service, Beltsville, MD 20705, USA; 2 School of Agriculture, Food and Wine, The University of Adelaide, Adelaide, SA 5005, Australia

Viroids were discovered by Diener in 1971 [1,2], and represent a third step in understanding the evolution of the biosphere following the initial visualization of microorganisms by Van Leeuwenhoek in 1675 and the first recognition of the submicroscopic world of viruses by Ivanovsky in 1892 [2]. This editorial acknowledges the work of the late Ricardo Flores of Spain, whose work with viroids over four decades has contributed to many discoveries in the field.


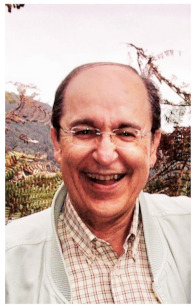


Flores (deceased 20 December 2020) was Research Professor of the Spanish National Research Council (CSIC) at the Institute of Plant Molecular and Cellular Biology, Polytechnical University of Valencia (UPV), Valencia, Spain. Born on 27 January 1947 in Almoradí (Alicante), Spain; he was schooled in the Jesuit tradition and graduated from UPV as an Agronomic Engineer, along with a BSc (Chemistry). He received his Ph.D. in Chemistry in 1975 under the supervision of Vicente Conejero for a thesis on citrus tristeza virus (the cause of citrus ‘quick decline’ epidemics in citrus worldwide). After his appointment at the CSIC in 1979, he spent 1981/1982 on sabbatical leave in the United States with Joseph S. Semancik at University of California, Riverside, CA, working on the properties of a cell-free system for the synthesis of citrus exocortis viroid (CEVd). In that system, linear and circular viroid molecules were synthesized by DNA-dependent RNA polymerase using the viroid RNA as template. This led to his research on viroids and, over the next four decades, he contributed groundbreaking research on viroid replication mechanisms (involving enzymes and ribozymes), molecular structure, pathogenesis mechanisms, evolutionary origin, and the discovery of the viroid etiology of four diseases of unknown cause.

He published more than 180 scientific papers and a number of book chapters. He was the major advisor to around 20 PhD students, many now publishing in both viroid and virus research. He hosted about 15 post-doctoral scientists and visiting scientists in his laboratory, attended international conferences as a keynote speaker, completed field studies when on international visits, and was a member of a range of national and international panels for evaluating scientists at universities and research institutes.

Skilled in editing, Dr. Flores was an associate editor or a member of the editorial board of the journals Frontiers in Microbiology, Frontiers in Plant Science, Viruses, Virus Research, Archives of Virology, RNA Biology, Heliyon, Journal of Plant Pathology and Molecular Plant Pathology. In addition, he was ad hoc reviewer for several US, Canadian, Australian and European journals that deal with articles on plant virology. He co-edited and contributed chapters in two books on viroids: Viroids, published in 2003 and edited by Ahmed Hadidi, Ricardo Flores, John W. Randles and Joseph S. Semancik; Viroids and Satellites, published in 2017 and edited by Ahmed Hadidi, Ricardo Flores, John W. Randles and Peter Palukaitis. Viroids was published by CSIRO Publishing with 370 pages. Viroids and Satellites was published by Academic Press/Elsevier with 716 pages.

Dr. Flores was past Chairperson (1993–2004) of the Viroids Study Group of the International Committee for Taxonomy of Viruses, and past Vice President of the Spanish Society for Virology (2007–2013). Among his honors and awards, he received the Biannual Award of the Spanish Society for Virology in 2003, became an Honorary Member of the Hungarian Academy of Sciences in 2008, and received the Plaque of Honor of the Spanish Association of Scientists in 2015.

Dr. Flores was a cultured gentleman, especially knowledgeable about his country’s history, hard-working, generous, kind and hospitable. He is survived by his wife Marita, their daughter María and their son Ricardo II, and he was a proud grandfather to his ‘little ladies’.

The novelty of viroids is that they do not encode proteins, using pre-existing host cell RNA polymerase and processing enzymes for replication and pathogenesis [1,2]. Members of the family *Pospiviroidae* replicate in host nuclei, whereas members of the family *Avsunviroidae* replicate in host chloroplasts [1,2]. In addition, one step of the replication cycle in the family *Avsunviroidae* is catalyzed by hammerhead ribozymes embedded in viroid strands.

Flores’s laboratory presented evidence that the replication of avocado sunblotch viroid (ASBVd) occurs through a symmetric pathway via two rolling circles with hammerhead ribozyme processing [3,4,5]. Furthermore, complexes containing both polarity strands of ASBVd were identified in chloroplasts of infected avocado plants and characterized [6].

Flores had shown, in a cell-free system, that the synthesis of CEVd (*Pospiviroidae*) was inhibited by alpha amanitin, an inhibitor of RNA polymerase II (Pol II) [7]. This finding suggested that CEVd alters the specificity of the enzyme because Pol II typically transcribes DNA. In contrast to CEVd, in vitro experiments with ASBVd showed that this viroid is insensitive to alpha amanitin and tagetitoxin. Tagetitoxin inhibits the plastid -encoded polymerase (PEP) but not the nuclear-encoded polymerase (NEP) [8]. Thus, NEP is possibly a good candidate for mediating the replication of ASBVd and other members of the family *Avsunviroidae*. This was supported by the replication of the peach latent mosaic viroid (PLMVd) calico variant in peach leaves that lack PEP [9]. NEP begins the synthesis of both polarity strands of ASBVd and PLMVd in specific sequence/structure motifs [10,11], as demonstrated by experiments that utilized a specific property of chloroplast transcripts.

Flores’s laboratory extended the original observation of the self-cleavage of plus and minus RNA transcripts of ASBVd by hammerhead ribozymes [12] to other members of the family *Avsunviroidae* [4]. It was demonstrated that the ribozyme activity is facilitated by tertiary contacts between apical loops [13,14] and interactions with host proteins that promote the adoption of the catalytically active conformation during transcription [15]. Flores’s laboratory also reported the in vivo processing to monomeric forms of dimeric plus transcripts of CEVd, hop stunt viroid, and apple scar skin viroid (family *Pospiviroidae*) in transgenic *Arabidopsis thaliana* [16]. The corresponding dimeric minus transcripts of the above three viroids were not processed. The cleavage sites of the dimeric plus the transcripts of the above three viroids have been mapped at similar positions of the capping loop of Hairpin I (HP1) [16]. Thus, the corresponding sequences of the dimeric or oligomeric RNAs would facilitate the adoption of an alternative long double-stranded region, as proposed previously [17,18], the actual substrate for cleavage. Cleavage at both strands of the double-stranded region leaves two 3′ terminal nucleotides, the fingerprint of RNases of class III. The monomeric viroid linear plus strands that accumulate in *A. thaliana,* expressing the corresponding dimeric transcripts, contain such terminal groups [19].

Moreover, Flores’s laboratory demonstrated that members of the genus *Pospiviroid*, family *Pospiviroidae,* redirect host nuclear DNA ligase 1 to act as an RNA ligase to circularize the viroid monomeric linear RNA in vivo [20]. The circularization is further continued by the rod-like secondary structure that, in its CCR, contains a local element of the tertiary structure, which positions the termini to be ligated in close proximity and in the correct orientation [16]. Ligation involves both the upper and lower CCR strands. Cleavage, however, is determined only by the upper CCR strand. The hammerhead-mediated self-cleavage in the family *Avsunviroidae* generates monomeric linear forms with termini specific to the wheatgerm tRNA ligase. Experiments with a recombinant of the chloroplast isoform of the tRNA ligase from eggplant showed the efficient circularization in vitro of the plus and minus monomeric linear replication intermediates of viroid members of the family *Avsunviroidae* [21].

For viroid structure, it was demonstrated by Flores’s group that: (1) both polarity strands of ASBVd, PLMVd, chrysanthemum chlorotic mottle viroid (ChCMVd), and eggplant latent viroid (ELVd) can adopt hammerhead structures that mediate their self-cleavage in vitro and in vivo during replication in plastids through a symmetric rolling circle [4]; (2) PLMVd and ChCMVd each have a multibranched secondary structure, stabilized in the plus strands by a kissing loop interaction [22,23,24]; (3) the prototype PLMVd variant has 338 nt [22,23], and the size of other variants vary from 335 to 351 nt [9,25]; (4) some of the PLMVd plus and minus linear forms from infected tissues are opened at the hammerhead-predicted sites [10]; (5) UV irradiation revealed additional elements of tertiary structure in the PLMVd plus strand [26]; (6) the initiation site for PLMVd plus RNA is at C51 and at U286 for the viroid minus RNA, with these sites mapping at similar double-stranded motifs of 6–7 bp that also contain the highly conserved GUC triplet preceding the self-cleavage site in both polarity strands [10]; (7) a single initiation site of the ASBVd plus strand has been mapped in vivo at position U121, while U119 is the single initiation site of the ASBVd minus strand and each site starts with the same sequence, UAAAA. Both sites, which are only two residues apart, are located in the A+U-rich right terminal loop of the viroid secondary structure [11]; (8) NEP begins the synthesis of both polarity strands of PLMVd and ASBVd in specific sequence/structure motifs [10,11]; (9) both strands of ELVd fold into a relatively long rod-like central domain that ends with a three-way junction in the left domain. The plus strand also possesses a three-way junction with three stems that form its right domain, whereas the right domain on the minus strand ends with five stems [27,28]; (10) in oligomeric PSTVd plus strands, Hairpin I (HPI) facilitates the adoption of a conserved double-stranded structure that is the substrate for their in vivo cleavage [16]; (11) PSTVd contains, in vivo, a loop E motif [29] located at the CCR, which is involved in multiple functional roles, particularly ligation [16].

Flores’s laboratory also studied viroid pathogenicity at the molecular level [30,31]. It was revealed that RNA silencing restrains viroid infection, as demonstrated by the detection and characterization of viroid small RNAs (vd-sRNAs) in tissues infected by PLMVd (member of the family *Avsunviroidae*) and potato spindle tuber viroid (PSTVd) (member of the family *Pospiviroidae*). These vd-sRNAs reduce, in a sequence-specific manner, the expression of specific genes and the viroid titer in infected plants. Moreover, RNA-directed RNA polymerase 6 reduces the titer of PSTVd and blocks its entry into meristems. These data, particularly the sequence-specific effects of vd-sRNAs, suggest that one or more Argonaute proteins (AGOs) at the core of the RNA-inducing silencing complex (RISC) are also involved. In addition to guiding RISC against viroid RNAs, vd-sRNAs could also target specific host mRNAs, whose inactivation would ultimately result in symptom development through a signal transduction cascade. It was demonstrated that certain variants of PLMVd containing a specific 12–14 nt insertion elicit an extreme albinism known as “peach calico”, which is triggered by two 21-nt PLMVd-sRNAs that are complementary to a 21-nt sequence in the peach mRNA coding for chloroplast heat shock protein 90 and target it for cleavage at the expected site (between positions 10 and 11 of the PLMVd-sRNAs).

The discovery that RNA can store genetic information and display catalytic activity suggested that RNA emerged before DNA and proteins. Due to the ribozyme activity of certain introns, considered a relic of the RNA world, Diener [32] proposed that viroids could also be regarded as relics of an ancient RNA world, and viroids were better candidates as “living fossils” of a precellular RNA world than introns. Diener [32] and Flores et al. [33] stated that the salient features of viroids that make them good candidates as survivors of the RNA world include those expected for primitive RNA replicons: (1) small size imposed by error-prone replication; (2) high G+C content to increase replication fidelity; (3) circular structure to assure complete replication without genomic tags; (4) structural periodicity for modular assembly into enlarged genomes; (5) lack of protein-coding ability consistent with a ribosome-free habitat; (6) replication mediated in some of them by ribozymes, the fingerprint of the RNA World. In the context of the error-prone replication of viroids, Flores’s laboratory demonstrated that the mutation rate of ChCMVd (*Avsunviroidae*) is the highest reported for any biological entity [24]. This extreme rate, which is presumably the consequence of the errors introduced by NEP when transcribing non-physiological RNA templates, and its lack of proofreading activity, is most likely tolerated because of the small size of the viroid genomes [24]. Phylogenetic reconstructions by Flores’s group suggest a monophyletic origin for viroids [34,35]. However, the possibility of a polyphyletic viroid origin cannot be dismissed, particularly considering that ASBVd is A:U rich, in contrast to the other viroids [33].

In addition, Flores’ research was instrumental in revealing the viroid etiology of four economically important plant diseases, namely peach latent mosaic, pear blister canker, apple dimple fruit and chrysanthemum chlorotic mottle [1,2,36].
